# Genetic parameter changes and age−age correlations in *Pinus koraiensis* growth over 40-year progeny testing

**DOI:** 10.1186/s12870-024-04752-y

**Published:** 2024-02-03

**Authors:** Kyungmi Lee, Changyoung Oh, In Sik Kim

**Affiliations:** Forest Tree improvement and Biotechnology Division, Forest Bioresources Department, National Institute of Forest Science, Suwon, 16631 Republic of Korea

**Keywords:** Genetic parameters, Age−age correlation, Progeny testing, Tree improvement, *Pinus koraiensis*

## Abstract

**Background:**

Early selection in tree breeding could be achieved by addressing the longevity of tree improvement activities. Genetic parameter changes and age–age correlations are essential for determining the optimal timing of early selection. Practical tracking of genetic parameters of *Pinus koraiensis*, a major timber species with economic and ecological value, has become feasible as its progeny testing has entered the mid-term age in Korea. However, research on the age−age correlation of *P. koraiensis* as progeny trials approach rotation age is limited. This study aimed to investigate genetic parameter trends and age−age correlations in *P. koraiensis* progeny. *P. koraiensis* progeny were assessed at two sites using a linear mixed-effects model with two-dimensional spatial autoregressive structure. Height, diameter, and volume growth were measured in 11 assessments over 40 years.

**Results:**

Genetic parameters, such as height and diameter, showed different patterns of change. The heritability ranged for the three growth traits in 0.083–0.710, 0.288–0.781, and 0.299–0.755 across the sites and age. Height heritability and its coefficient of variance decreased, whereas the diameter and volume estimates remained relatively constant. Correlations with Age 40 for phenotypic, genetic, and rank of breeding values ranged between 0.16 and 0.92, 0.594 and 0.988, and 0.412 and 0.965, respectively. These correlations generally increased as the age approached Age 40, with particularly high levels observed at Age 26 and Age 30.

**Conclusion:**

The observed genetic trends in *P. koraiensis* progeny testing offer valuable insights for early and precise selection. Notably, selecting superior genotypes at Ages 26–30 is supported by discernible genetic gains and robust correlations. Future research should integrate unbalanced data for selecting mother trees or families and conduct a comprehensive economic analysis of early selection to validate its practical benefits.

**Supplementary Information:**

The online version contains supplementary material available at 10.1186/s12870-024-04752-y.

## Background

The genetic improvement of long-lived trees aims at the gradual enhancement of population traits through recurrent selection and testing [1]. Modern forest tree improvement programs, initiated worldwide in the 1950s, have persisted in a continuous cycle of breeding, testing, and selection [2]. Progeny trials is an essential step in tree improvement programs [3, 4]. They involve evaluation of breeding materials based on the performance of their offspring, which are selected recurrently for advanced generations. A major challenge in conducting precise and efficient progeny testing of tree breeding materials arises from the longevity of tree species [5, 6]. The long lifespan of tree species necessitates prolonged progeny trials, which can pose a direct hindrance to tree improvement programs. This challenge is particularly notable in the context of temperate conifer species, which typically have an optimum rotation age of 40 to 60 years [3]. It is widely recommended to conduct progeny trials for a minimum of half the rotation age [4, 7].

Early selection has been attempted to minimize tree improvement time and cost [8]. The age−age correlation is important criterion in early selection as it allows for the prediction of genetic gain at the optimal rotation age based on genetic gain at an earlier age [3]. However, age−age correlation remains a challenge for precise early selection in efforts to enhance the economic feasibility of tree improvement [9]. To determine the optimal selection age, it is necessary to examine genetic parameters, including variance, heritability, and age−age correlations of the target traits [10]. The importance of understanding age−age correlations is also emphasized by the inconsistency in performance in the early ages of tree growth [4].

*Pinus koraiensis*, commonly known as Korean pine, is a major timber species with economic and ecological value in the cold-temperate forests across northeast Asia and far-east Russia [11–13]. Its robust trunk is prized in construction, shipbuilding, and furniture, making it a vital native economic species [13]. The seeds, rich in fats, proteins, and carbohydrates, serve as a valuable resource for oil extraction and nutrition [12, 13]. Pine seed oil from Korean pine is linked to blood lipid regulation [13]. Additionally, these forests positively impact the environment, regulating temperature, humidity, water, and wind speed, while their extensive root systems stabilize soil and control erosion [13]. An intensive tree improvement program for *P. koraiensis* was initiated in South Korea in the 1960s [14]. 300 trees were selected as plus trees from the natural distribution range of the species; subsequently, 10 sets of half-sib progeny trials were established from 1975 to 1994 using open-pollinated progeny from plus trees. Each set consisted of one to three replicates. Seed orchards for the species were established on 101 ha of land between 1965 and 2015 (50 years), and seed production of 9,260 kg has been achieved annually over the last five years from orchards [15].

Despite intensive tree improvement of *P. koraiensis* not only in Korea but also in Northeast China [12], the advancement of the breeding cycle is slow owing to its long breeding cycle [16]. Research on the age−age correlation of *P. koraiensis* as progeny trials approach rotation age is limited. Insufficiency of long-term progeny testing data is rather common in tree improvement, and the accumulated data rarely exceed 35 years, even in pine species [4]. Age−age correlation studies with genetic parameter based on practical data of multidecadal investigations are rare today, although there are a few studies that have applied prediction models [3, 9] or growth data per se [4].

Therefore, the objectives of the present study were to investigate the: (1) growth and genetic parameter changes over time, and (2) age−age correlation of tree growth in 40-year-old progeny trials of *P. koraiensis*. The findings of the present study could facilitate early selection of *P. koraiensis* individuals in progeny trials, as well as tree breeding activities and forest management in general. It is noteworthy that this study analyzed the age−age correlation based on tracking actual tree growth for 40 years.

## Results

### Genetic testing based on growth performance data from a progeny trial at Age 40

Average value of height, diameter, and volume of *P. koraiensis* were significantly different between the two sites, with greater growth in all traits at CJ (Table 1; *p* < 0.0001 for height and volume, and *p* < 0.05). The average height, diameter, and volume values were 17.2 m, 21.78 cm, and 0.3414 m^3^, respectively, at CJ; in contrast, the average values at GP were 13.3 m, 21.22 cm, and 0.2482 m^3^, respectively, showing significantly lower growth than that at CJ.

**Table 1:** Number of assessments and ages evaluated in the *Pinus koraiensis* progeny trial of 1985

Site	CJ	GP
Number of planted trees	1,458	1,154
Number of remained trees	997	824
Number of assessments	11	12
Age of growth trait assessment	Age 5, 8, 10, 13, 15, 18, 20, 23, 26, 30, 40	Age 5, 8, 10, 15, 18, 20, 23, 26, 29, 30, 35, 40

**Fig. 1:**
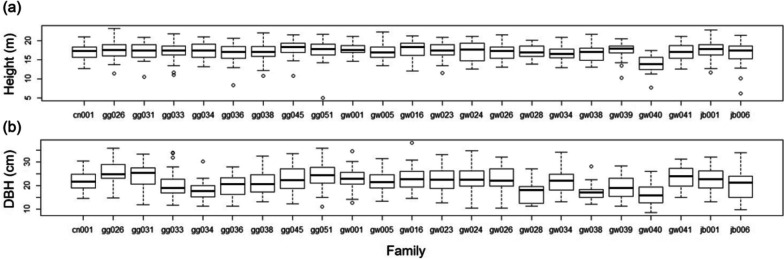
Boxplots of (**a**) height and (**b**) diameter of the *Pinus koraiensis* progeny trial in CJ

The estimated heritability of volume at the latest investigated age of 40 years was 0.633 and 0.419 in CJ and GP, respectively (Tables S1 and S2). The estimates were higher than those (0.490 and 0.406, respectively) obtained using a general linear mixed-effects model with family, block, and their interaction effects in the same dataset. The heritability estimates for height and diameter at Age 40 were 0.139 and 0.769, respectively, at CJ, and 0.083 and 0.472, respectively, at GP. The heritability estimate for diameter was unusually high in CJ, and the height and diameter data distribution based on family were examined using boxplots (Fig. 1). The median values and their interquartile ranges were generally more discrete for diameter than for height based on family.

### Genetic parameter changes with age and age−age correlation

**Fig. 2:**
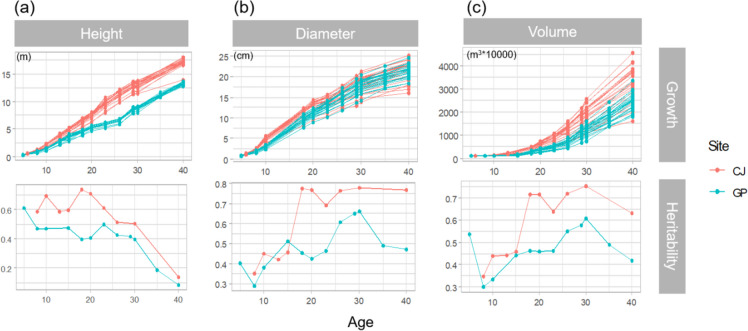
Changes in growth and heritability for (**a**) height, (**b**) diameter, and (**c**) volume by age in the *Pinus koraiensis* progeny trial

Here, height, diameter, and volume growth of *P. koraiensis* within each family were investigated over a 40-year period (Fig. 2). Height growth continued until Age 40, while diameter growth showed a tendency to slow down from Age 30. Although growth differences between sites were significant for all traits (Table 1), height exhibited a more pronounced difference between sites than diameter, as trees showed complete separation between sites regardless of family type.

Heritability varied with age (Fig. 2; Tables S1 and S2). At all assessed ages, the heritability estimates for the three traits were mostly higher in CJ than in GP. Height heritability was relatively high before Age 23 and decreased thereafter, whereas diameter heritability was highest at Age 30 at both sites, showing a general increase with age. Volume heritability exhibited a pattern similar to diameter heritability, with values of 0.633−0.755 recorded in CJ from Age 18, which is a markedly high value for tree growth. The estimates decreased from Age 30 at both sites, but were maintained at considerable levels of 0.633 and 0.419 in CJ and GP, respectively.

**Fig. 3:**
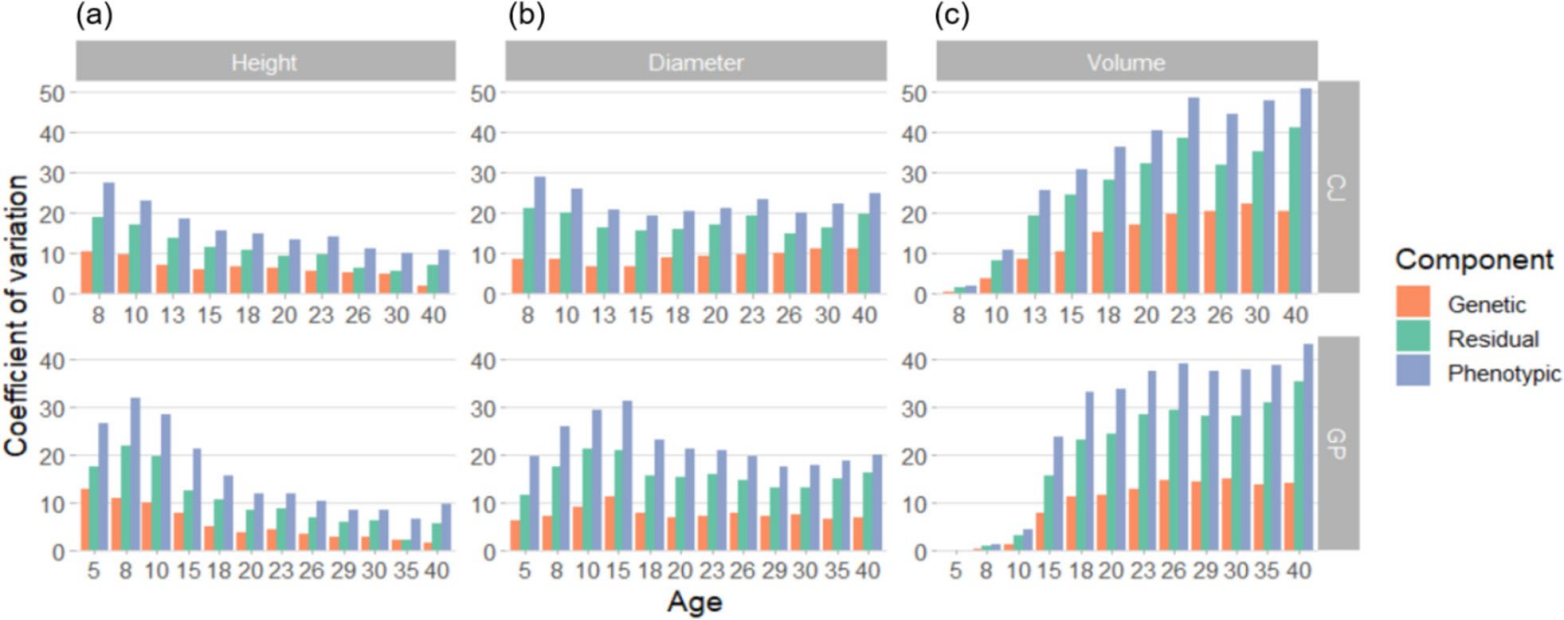
Coefficient of variation of genetic, phenotypic, and environmental factors for (**a**) height, (**b**) diameter, and (**c**) volume in *Pinus koraiensis* at two trial sites (CJ and GP).

The *CVs* of the genetic, phenotypic, and environmental factors for height, diameter, and volume were estimated (Fig. 3). The trends differed according to trait. The *CV*_*G*_ and *CV*_*P*_ decreased in the case of height, whereas they were maintained in the case of diameter, with aging. The *CV* of volume, which was derived by the product of height and diameter squared, exhibited an increasing trend with growth. At CJ, the *CV*_*G*_ of height decreased from 10.56% at Age 8 to 2.04% at Age 40 with growth. Similarly, the *CV*_*P*_ of height decreased from 27.48% at Age 8 to 10.90% at Age 40. For diameter, *CV*_*G*_ increased from 8.61% at Age 8 to 11.04% at Age 40, and *CV*_*P*_ was maintained in the 19.44−28.94% range over 40 years. The *CV*_*G*_ of volume was 0.62% at Age 8 and 20.47% at Age 40. Both *CV*_*G*_ and *CV*_*P*_ of volume were relatively constant from approximately Age 23 at the two sites. The trends were similar at the two sites, whereas the estimates at GP were generally lower than those at CJ.

**Fig. 4:**
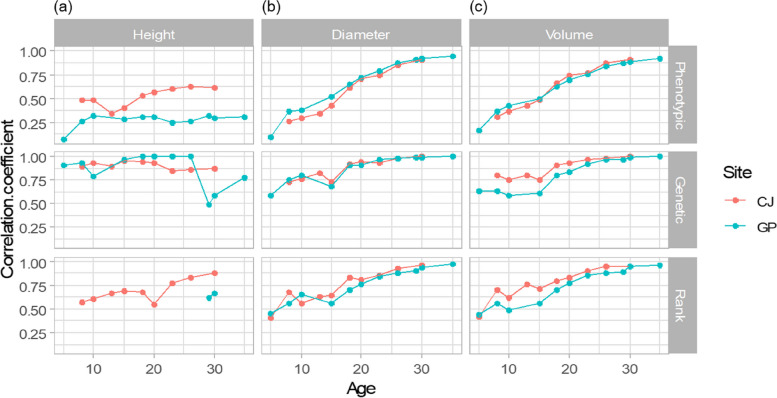
Correlation coefficients of volume between all ages and Age 40 for phenotypic, genetic, and Spearman’s rank correlation for (**a**) height, (**b**) diameter, and (**c**) volume in the *Pinus koraiensis* progeny trial

The phenotypic correlation between each age and Age 40 was relatively constant for height when compared with diameter and volume (Fig. 4a). The latter two exhibited increasing trends as age approached 40. The height correlation was stronger in CJ than in GP, whereas the correlations of diameter and volume were similar at both sites. The phenotypic correlation of volume between Age 8 and Age 40 was low, at 0.31 and 0.37, in CJ and GP, respectively. It increased to 0.5 at Age 15, 0.77 at Age 23, and was approximately 0.9 (0.91 and 0.89) at Age 30 at both sites.

The genetic correlations of height between all age groups and Age 40 were generally much stronger than the corresponding phenotypic correlations (Fig. 4b). The genetic correlation of diameter showed an increasing trend, similar to phenotypic correlation, but started at a higher value. The level of genetic correlation for volume was rather distinct between the two sites until Age 20. The genetic correlation coefficients with Age 40 were 0.80 and 0.63 at the CJ and GP sites, respectively, at Age 8. It increased continuously, reaching 0.90 and 0.80 at CJ and GP, respectively, at Age 18.

Spearman’s rank correlation of height was estimated between all ages and Age 35 in GP, as the model analysis for estimating the breeding value of the plus trees at the site did not converge at Age 40 (Fig. 4c). The rank correlation with Age 35 was significant only at Age 29 in the GP group. At CJ, the Spearman’s rank correlation generally increased for diameter and volume. For volume, the Spearman’s rank correlation was approximately 0.4 at Age 5, and it was approximately 0.8 and 0.9 at Age 20 and Age 26, respectively. The coefficient was 0.95 and 0.94 in CJ and GP, respectively, at Age 30.

**Fig. 5:**
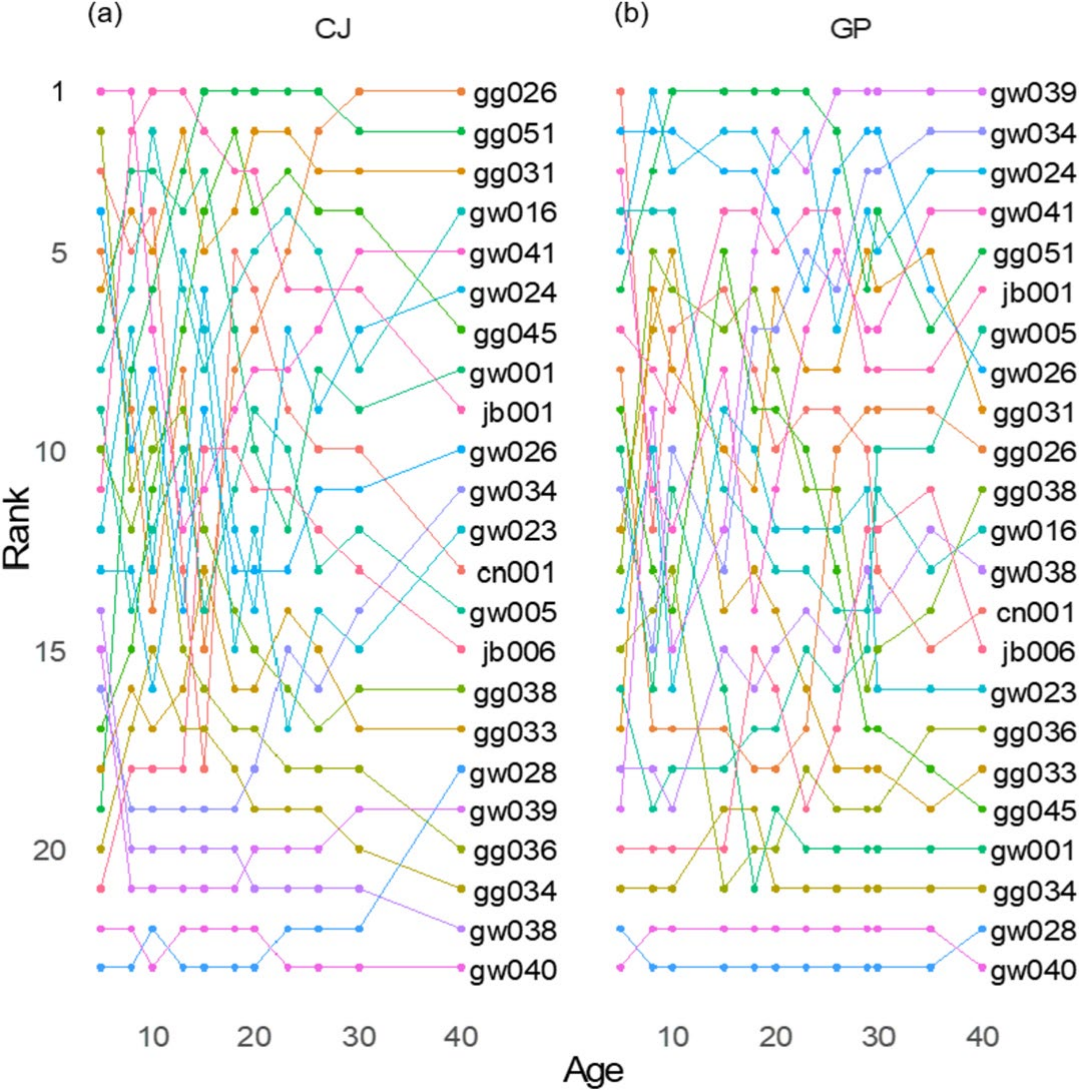
Breeding value rank trends of *Pinus koraiensis* plus trees by age in (**a**) CJ and (**b**) GP.

Specifically, the ranking trends of the breeding value of volume was determined to identify the actual trends according to age (Fig. 5). Fluctuations in ranking were relatively weaker in families with a low breeding value. Notably, gw040, gg028, and gg034 consistently exhibited low breeding values at both sites. The ranking of volume showed different patterns in the two sites based on family at Age 40. Among the 23 families with an upper rank at age 40, gw026, gg051, gw041, and gw024 were superior at both sites. However, gg045 and gw001, which had the 7th and 8th highest breeding values in CJ, respectively, had the 19th and 20th highest breeding values in GP, respectively, at Age 40. Similarly, gw039 exhibited the highest breeding value in GP but the 19th highest breeding value in CJ at Age 40.

## Discussion

### Genetic testing based on growth performance data in 40-year progeny trial

In the present study, *P. koraiensis* growth patterns were investigated as a prerequisite for formulating tree improvement programs and early selection of long-lived conifer tree species [17]. Growth pattern information is vital for the prediction of biomass production and understanding of adaptability trends [17, 18]. Within the mid-level range of the three site index levels (12, 14, 16) in South Korea, the average growth of *P. koraiensis* forest stands at age 40 is 15.1 m in height and 27.6 cm in DBH, with a density of 516 trees per hectare, from the empirical yield table for the species [19]. Here, height growth was significantly different between the two study sites, with superior growth in CJ when compared with that in GP. The height of the dominant trees in CJ was approximately 20 m, with the highest site index of 16 (19.4 m) achieved in the empirical yield table for *P. koraiensis* in Korea [19]. The average height of 17.2 m in CJ also corresponded to the site index of 16. In contrast, the height of the dominant trees in GP was approximately 18 m, which corresponded to a site index of 14. However, the average height in GP was only 13.3 m, failing to reach the empirical average value of site index 14 (15.1 m). The average height in GP corresponded to site index of 12 (13.2 m) in terms of average height growth. The estimation of the site index of the two sites was used to determine the general growth status of the progeny in the trial site although the stand density in this study differed from the empirical yield table. Additionally, there was potential overestimation of site index as the experimental plantation consisted of the progeny of plus trees. Diameter and volume growth were also larger in CJ than in GP, with the average DBH values of 21.78 cm and 21.22 cm. These average at both sites were much lower than the 26.7 cm observed in the lowest site index of 12.

Although forest management records were not available, the delayed DBH growth in the present study when compared with that in the general stand was thought to be due to tree density. The experimental trees were initially planted at intervals of 1.8 m and thinning generally resulted in intervals of 3.6 m without the natural death of trees. In the 35-year-old *P. koraiensis* progeny testing study with different open-pollinated families in CJ and GP, the average heights were 12.7 m and 10.9 m, respectively [11]. The average diameters of 19.3 and 20.3 cm in CJ and GP, respectively, showed similar levels of growth in the present study, although DBH growth was greater in GP than in CJ [11], unlike in the present study. The difference in volume growth between the two sites was not significant in the previous study. Local heterogeneous environments within mountainous sites due to rugged topography could be one of the reasons for the complex growth performance. Topographic variation within forests can cause different tree growth performance, even at the fine scale, by affecting site parameters [20]. The growth characteristics of *P. koraiensis* in the progeny trial revealed in the present study could serve as additional examples to facilitate the understanding of *P. koraiensis* growth at the landscape scale in the Northeastern Asia environment. By extension, different patterns from the same region highlight the necessity for additional case analyses to understand *P. koraiensis* growth performance.

The linear mixed model in this study included family effects as genetic and spatial effects with an autoregressive structure were treated as environmental. However, a limitation of the model exists in not considering other random effects, specifically block and individual tree effects. There is a possibility of differences in stand density between blocks, which can influence tree growth. Individual tree effects are present as measurements were repeated for each tree, even though analyses were conducted separately by age. Attempts to include detailed random effects, such as block, individual, and the interaction between family and block, did not result in a clear improvement in fit in most analyses. This inconvergence or lack of improved fitness in models with detailed random effects is thought to result from model complexity [21] or the data structure with one measurement for each individual at each age, which is common in progeny testing in timber trees. Thus, a unified model with what can be considered as basic effects was unavoidably used to examine genetic effect changes across ages.

This study revealed intermediate to high levels of heritability of volume in *P. koraiensis*. The remarkable estimate is consistent with that of previous studies on progeny testing in the species. Individual heritability was 0.73 in 20-year *P. koraiensis* height growth [22], and ranged from 0.566 to 0.681 in a full-sib progeny trial [23]. In addition, the estimates ranged from 0.910 to 0.990 in a full-sib progeny trial of the species [24]. This high heritability in the present study is consistent with the results of another study [25]. Heritability estimates for height, DBH, and volume were 0.439, 0.746, and 0.745, respectively, in half-sib progeny trials [26]. The high level of heritability in the studies revealed a strong selection effect on the species [24, 26]. The potential interpretation of considerable heritability observed in some studies of *P. koraiensis* was not found in the existing literature. In this study, the growth difference by family, especially notable between the extreme families, was supposed to confirm the significant heritability. For example, the discrete distribution of measurements by family was remarkable in the DBH of gg026 and gw038 (Fig. 1). However, a recent study involving another progeny trial of the species at the sites showed moderate heritability [11], which was lower than the estimates in the present study. Although the analysis in the previous study was performed using an linear mixed-effects model, the different levels of the parameters showed significant differences across trial or local environment, even for the same species and region. Genetic parameters can change as trees develop and vary largely with age, microsite, spacing, and environmental stress [27]. The variability in genetic parameters highlights the importance of quantifying the genetic effects on target traits to assess field trials. That is, heritability can be considered an index for understanding the efficiency of a specific field trial set rather than a constant value [27].

### Genetic parameter changes with age and age−age correlation

In the present study, height differences across sites were consistently significant from Age 08, confirming the different site indices of the two sites. CJ was considered to have more favorable site conditions based on the site index inferred by height growth. The higher heritability across ages in CJ than in GP indicates that genetic effects are more distinguishable under favorable environmental conditions [11, 28]. Volume heritability in GP changed in a similar pattern but had generally lower values than those in CJ. The highest volume heritability observed at Age 30 in the present study suggests that this is a feasible period for selecting superior genotypes. The varied heritability patterns by age, commonly observed at the two sites, were remarkable, despite the different levels at the sites. At both sites, height heritability was higher than diameter heritability at the early age. However, the former declined, whereas the latter increased changing the order of estimates for the two traits. The significant height difference across sites, coupled with its decreasing genetic effect, suggests a strong influence of environmental factors on tree height, contrary to the diameter growth pattern. However, in a previous study in the same region, a higher heritability for height than for DBH was observed at Age 35 [11].

Drawing a generalized conclusion about heritability changes with age is cumbersome. The temporal pattern of heritability varies by case. In some studies, height heritability increases with age; while in others, it decreases, or no clear pattern exists [27]. In addition, height heritability shows different patterns at different sites within the same trial [29]. The height heritability at one site was maintained for approximately 16 years, as reported in some studies [25, 30]; however, it decreased with age at other sites [29], as reported in a previous study [31]. A decrease in height heritability with an increase in diameter heritability was revealed in an *Abies alba* study [32]. In the present study, heritability was thought to decrease with an increase in age due to increased noise, including the competition effect.

In addition to microsite variance, family competition can affect heritability. Intense competition between families can increase heritability [33]; however, the effect can be delayed in trials with multi-tree plots. Inter-tree competition decreases family effects with age [34]. Intensification of growth and decline in heritability was observed in some conifer species, which was related with “Mature Genotypic Phase” [32, 33]. While the lower heritability in height compared to diameter has sparked debates concerning the relative susceptibility of height to tree density in comparison to other traits [27], substantial heritability in diameter is interpreted as indicative of a stronger genetic influence on this trait [32]. The different patterns in height and diameter in the present study were thought to show moderate competition, which was not high enough to cause the same trends, regardless of trait.

The *CV* trends differed across traits as trees grew. The genetic and phenotypic *CV*s decreased with height, whereas the estimates were maintained for diameter. In the case of volume, which was derived by multiplying height and diameter squared, *CV* was also maintained at a substantial level at both sites. The higher *CV* in diameter than in height was consistent with the findings of previous progeny testing of *P. koraiensis* in Korea [11]. This can be explained by the larger genetic variability and broader scope of selection for diameter than for height. Considering the greater genetic effect on diameter than on height, diameter selection can be more effective in this case, with higher heritability and larger genetic variation indicating evolvability [35].

The age−age correlation of phenotype, genotype, and rank of estimated breeding values of height, diameter, and volume with Age 40 were analyzed at all ages. All correlations increased gradually as the trees approached a base age 40. In the case of volume, the phenotypic correlation with Age 40 started at approximately 0.3, whereas the genetic correlation began > 0.6. The phenotypic correlation increased continuously; however, the genetic correlation became relatively flat from Age 26 at both sites, resulting in a coefficient > 0.95. Our results show that the underlying genetic correlation between age was stronger than the phenotypic correlation, as reported in previous studies [36, 37]. The changes in Spearman’s correlation with Age 40 also tended to be low after Age 26, with a coefficient > 0.85. As phenotypes and ranks are the actual indices of forest tree improvement, we concluded that selection after Age 26 can prove to be more effective than before. In a previous study on white spruce and lodgepole pine, the optimum selection age (OSA) was 40–50 years with an optimum rotation age (ORA) of 100 years [3]. In Zobel and Talbert (1984) [7], OSA was coincidentally similar to half-ORA. In the present study, the genetic correlation coefficient and Spearman’s rank correlation trends were relatively flat at Age 30, which was half the rotation age of the species in the national forest. Although last observation in this study was at Age 40, the pattern is expected to be maintained, assuming a higher correlation between half age and increased rotation age in general [1]. The actual genetic and phenotypic correlation can be utilized to predict the genetic gain at rotation age based on measurements taken at a younger age [38]. Consequently, early selection can be implemented to reduce the generation interval and maximize the gain per year in forest tree improvement [10]. However, a cost comparison between waiting until a certain age and earlier selection with potential errors is required to determine the efficiency of the selection precisely.

The breeding value ranking, in terms of volume, changed with age (Fig. 5). Although the trait value was used for ranking instead of the breeding value, a recent study suggested that there could be a discrepancy in high-performing families with age [4]. However, the results showed better ranking conformity over time, supporting early selection [29, 39, 40]. In the progeny testing of loblolly pine [40], the correlation of mean family volume showed few differences between 5 years and 15- or 20-years, resulting in similar levels of gain efficiency. Considering the various results and interpretations of performance over time, it would be appropriate to regard performance ranking stability as an indicator, similar to heritability, of characteristics of progeny testing. In the present study, ranking changes over time were caused by random environmental effects combined with various expression patterns of genetic factors [4]. In agreement with Bragg [4], this study supports the need to consider long-term performance when selecting for long-term wood production. However, further studies on the genetic effect changes on target traits over time are required, as there are few long-term studies covering the near rotation age. In addition, less fluctuation was observed in the lower-ranking families, indicating a stronger consistency of inferiority than superiority of volume growth in the present study. Notably, the breeding value ranks of inferior families, such as gw040, gg028, and gg034, remained stable at both sites. The exclusion of inferior families can increase genetic gain in a more assured manner than does selection of a few superior families with ranking variation based on age. This is supported by the cumbersome selection process for superior trees based on age and site.

## Conclusion

The present study provides notable insights that could facilitate *P. koraiensis* forest management and tree improvement. The results highlight the varying genetic effects of age on the growth of *P. koraiensis* and suggests that selecting superior genotypes at Age 26–30 is acceptable, given the observed genetic gain indicated by the Spearman’s correlation of breeding value and supported by the phenotypic and genetic correlations. This study unveils the shifts in genetic effects on growth and age–age correlations, drawing from the realized performance of progeny testing in *P. koraiensis*. These findings not only inform the determination of the optimal selection age (OSA) but also enable accurate predictions of genetic gain at rotation age, showcasing the innovative potential of this research. However, practical selection should consider phenotypic expression across multiple sites, ages, and timber harvesting times. Further research is needed to evaluate the economic effects of early selection by estimating the differences in genetic gain by age. Therefore, future studies should focus on increasing the number of sites and genotypes used as a way of improving the accuracy of the results. Furthermore, the interaction between genotype and environment should be studied to identify superior genotypes. Overall, the findings of this study enhance our understanding of *P. koraiensis* growth and genetic parameters and can be used to improve *P. koraiensis* forest management and tree breeding activities.

## Materials and methods

### Study sites, growth trait data assessment, and treatment

**Fig. 6:**
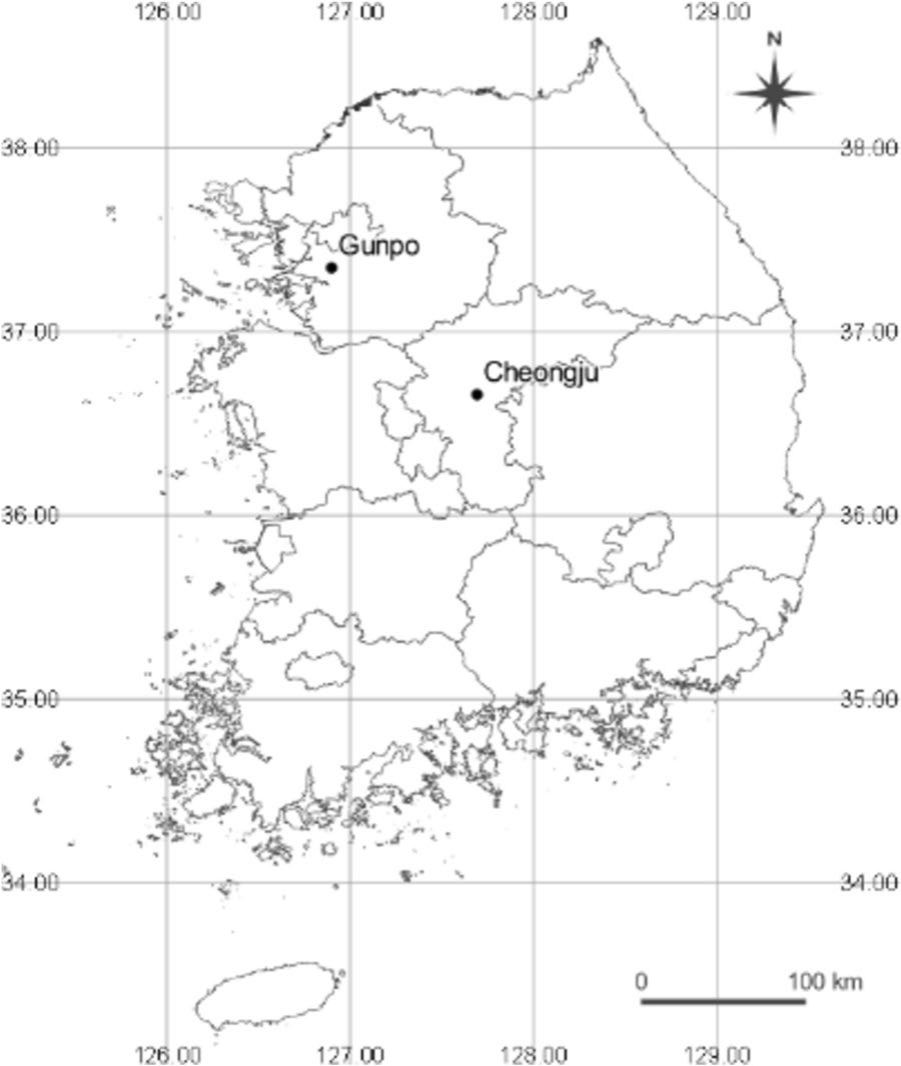
Locations of the two study sites (CJ and GP) in the *Pinus koraiensis* progeny trial established in 1985

The study site was a *P. koraiensis* progeny trial forest established in 1985. It consisted of 23 half-sib families with 6 blocks at 2 sites (Cheongju [CJ] and Gunpo [GP]), and the range of each area was 0.9 ha (Fig. 6). CJ was situated at 36°39’24"N 127°41’25"E, with an approximate altitude of 335 m. The mean annual temperature and rainfall were recorded as 13.1 ℃ and 1232.4 mm, respectively. GP, on the other hand, was positioned at 37°20’56"N 126°53’50"E, with an altitude of approximately 146 m. Its mean annual temperature and rainfall were reported as 13.3 ℃ and 1326.6 mm [41]. The numbers of trees planted in CJ and GP in 1985 were 1,458 and 1,154, respectively, and the numbers of trees remaining in 2020 were 997 and 824, respectively (Table 2). There were 957 and 785 experimental trees, respectively, after excluding trees in the control group.

**Table 2:** Average height, diameter, and volume indexes at two sites in the *Pinus koraiensis* progeny trial

Site	Number of trees	Height (m)	Diameter (cm)	Volume index (m^3^ × 10^4^)
Whole	1,742	15.4$\pm$0.1	21.53$\pm$0.12	0.2994$\pm$0.1525
CJ	957	17.2$\pm$0.1 a	21.78$\pm$0.17 a	0.3414$\pm$0.1707 a
GP	785	13.3$\pm$0.0 b	21.22$\pm$0.15 b	0.2482$\pm$0.1064 b

Growth surveys, including height and diameter measurements, were conducted 11 times at CJ and 12 times at GP over 40 years. The surveys were conducted in the same year, except at Age 13, Age 29, and Age 35. Age 13 data were available only for CJ, whereas Age 29 and Age 35 data were available only for GP. Growth characteristics were investigated manually for all ages except Age 40 when the forest survey was conducted precisely based on LiDAR using Trimble X7 3D Scanner equipment and Trimble RealWorks (Trimble Inc., Westminster, CO, USA). At Age 40, tree growth was scanned on 191 and 254 points in CJ and GP, respectively, on 0.9 ha plots, with matching errors of 4.90 and 4.97 mm, respectively. The volume of each tree was calculated based on height and diameter measurements [42].

$$Volume=0.01097+0.00003772{DBH}^{2}\times Height$$

In the analyses of genetic parameter changes and age−age correlations, volume was transformed by a factor of 10,000, as the actual value was minimal when the samples were seedlings. For the spatial distribution analysis of phenotype, which is required for the linear mixed-effects model analysis considering spatial effects, each individual tree’s location was assigned relative x and y coordinates, as they were initially planted in a grid at intervals of 1.8 m.

### Genetic testing of growth trait considering spatial phenotypic distribution

Differences in tree height, diameter, and volume between sites were analyzed using the Student’s t-tests. Genetic testing was conducted on the height, diameter, and volume growth traits at each site and each age under investigation. The growth data were analyzed using a model that separated the environmental effects by spatially dependent residuals based on an AR1(ρ_col_)⊗AR1(ρ_row_) structure. The spatial autoregression structure has been shown to be useful in explaining the spatial distribution of phenotypes in progeny trial studies [43–45]. The model is useful for considering the spatial autoregressive effect, which is usually observed in forest environments owing to biotic and abiotic differences at the micro site scale [44] complementing the block design on a broader scale. The model has been used to enhance genetic testing in tree improvement [43, 45–47].

The model is defined as:

$$\mathrm y=\mathrm{Xb}+\mathrm{Zu}+\mathrm\xi+\mathrm\eta$$

where y is the vector of measured data for each growth traits of height, diameter and volume, b is a vector of fixed effects of the grand mean with its design matrix X, u is a vector of random effects by family with it design matrix Z, ξ is spatially dependent residuals with a variance−covariance matrix of ${\sigma }_{{\upxi }}^{2}$[AR1(ρ_col_)⊗AR1(ρ_row_)], and η is spatially independent residuals [45, 47]. AR1(ρ_col_) and AR1(ρ_row_) are the first-order autoregressive correlation matrices in the column and row, respectively. Genetic testing using this a linear mixed-effects model with two-dimensional spatial autoregressive structure was performed using the R program [48] and the breedR package [49]. The heritability of each age group was estimated using the breedR package, and the breeding values of 23 families were estimated based on the random effects in the model at each age.

### Genetic parameter changes and age−age correlation with tree age

The growth patterns of height, diameter, and volume of *P. koraiensis* progeny were investigated based on assessment results over 40 years. As the root diameter and diameter at breast height (DBH) measurement were mixed depending on the site between Age 13 and Age 15, diameter measurement value during the period was omitted from the analysis. To examine changes in genetic parameters, the narrow-sense heritability and coefficient of variation (*CV*) of genetic (*CV*_*G*_), environmental (*CV*_*E*_), and phenotypic (*CV*_*P*_) factors were estimated using the R program [48] and the breedR package [49], as follows:

$${CV}_{G}=\frac{{\widehat{\sigma }}_{G}}{\stackrel{-}{X}}\times 100\%$$

$${CV}_{E}=\frac{{\widehat{\sigma }}_{E}}{\stackrel{-}{X}}\times 100\%$$

$${CV}_{P}=\frac{{\widehat{\sigma }}_{P}}{\stackrel{-}{X}}\times 100\%$$

To investigate the correlation between each analyzed age and Age 40, the phenotypic and genetic correlations as well as the Spearman’s rank correlation between growth traits at Age 40 and each of the other ages were analyzed. The ranks of the estimated breeding values of each family at Age 40 and the other ages were used to calculate Spearman’s rank correlation. The analyses were performed using R [48], PerformanceAnalytics [50], and the breedR package [49].

The phenotypic correlation coefficient (${r}_{{p}_{xy}}$) was calculated using the following equation:

$${r}_{{p}_{xy}} = \frac{{\sigma }_{{p}_{xy}}}{\sqrt{{\sigma }_{{p}_{x}}^{2}{\sigma }_{{p}_{y}}^{2}}}$$

where ${\sigma }_{{p}_{xy}}$ is the covariance between phenotypic effects for a trait at different ages; ${\sigma }_{{p}_{x}}^{2}$ and ${\sigma }_{{p}_{y}}^{2}$ are the estimated phenotypic variance for the trait in each age.

The genetic correlation coefficient (${r}_{{g}_{xy}}$) was calculated using the following equation:

$${r}_{{g}_{xy}} = \frac{{\sigma }_{{g}_{xy}}}{\sqrt{{\sigma }_{{g}_{x}}^{2}{\sigma }_{{g}_{y}}^{2}}}$$

where ${\sigma }_{{g}_{xy}}$ is the covariance between genetic effects for a trait at different ages, ${\sigma }_{{g}_{x}}^{2}$ and ${\sigma }_{{g}_{y}}^{2}$ are the estimated genetic variance for the trait at each age. In the present study, *x* was fixed at Age 40 for both correlation coefficients.

### Supplementary Information

**Additional file 1: Table S1.** Genetic and phenotypic coefficients of variation and heritability according to age in Chungju (CJ). **Table S2.** Genetic and phenotypic coefficients of variation and heritability by age in Gunpo (GP). **Table S3.** Phenotypic and genetic correlations of volume between Age 40 and other ages in the *Pinus koraiensis* progeny trial.

## Data Availability

The datasets supporting the conclusions of this article are included within the article and its additional file.

## Abbreviations

CJ: Cheongju
GP: Gunpo
DBH: Diameter at breast height
OSA: Optimum selection age
