# Supplementary material for: Genetic parameter changes and age−age correlations in Pinus koraiensis growth over 40-year progeny testing
Source: BMC Plant Biol. 2024 Feb 3;24:86. doi: 10.1186/s12870-024-04752-y (PMC10837979; doi:10.1186/s12870-024-04752-y)
Supplement: Supplementary file 1 — Additional file 1: Table S1. Genetic and phenotypic coefficients of variation and heritability according to age in Chungju (CJ). Table S2. Genetic and phenotypic coefficients of variation and heritability by age in Gunpo (GP). Table S3. Phenotypic and genetic correlations of volume between Age 40 and other ages in the Pinus koraiensis progeny trial. [file 12870_2024_4752_MOESM1_ESM.docx]

**Supplementary Material**

**Supplementary tables**

**Table S1.** Genetic and phenotypic coefficients of variation and heritability according to age in Chungju (CJ)

|  | Height | | | | Diameter | | | | Volume | | | |
| --- | --- | --- | --- | --- | --- | --- | --- | --- | --- | --- | --- | --- |
|  | *CV_G_* | *CV_P_* | *CV_E_* | *h^2^* | *CV_G_* | *CV_P_* | *CV_E_* | *h^2^* | *CV_G_* | *CV_P_* | *CV_E_* | *h^2^* |
| Age08 | 10.56 | 27.48 | 18.97 | 0.585 | 8.61 | 28.94 | 21.13 | 0.352 | 0.62 | 2.09 | 1.52 | 0.347 |
| Age10 | 9.75 | 23.25 | 17.10 | 0.694 | 8.75 | 26.00 | 20.06 | 0.449 | 3.62 | 10.86 | 8.19 | 0.440 |
| Age13 | 7.18 | 18.69 | 13.86 | 0.585 | 6.78 | 20.87 | 16.51 | 0.419 | 8.58 | 25.74 | 19.28 | 0.441 |
| Age15 | 6.10 | 15.74 | 11.55 | 0.594 | 6.61 | 19.44 | 15.65 | 0.458 | 10.55 | 31.00 | 24.45 | 0.459 |
| Age18 | 6.70 | 14.94 | 11.01 | 0.740 | 9.03 | 20.37 | 15.91 | 0.774 | 15.46 | 36.34 | 28.31 | 0.715 |
| Age20 | 6.29 | 13.33 | 9.43 | 0.710 | 9.35 | 21.20 | 17.11 | 0.767 | 17.21 | 40.42 | 32.50 | 0.715 |
| Age23 | 5.70 | 14.33 | 9.67 | 0.612 | 9.87 | 23.62 | 19.21 | 0.690 | 19.62 | 48.82 | 38.53 | 0.638 |
| Age26 | 5.42 | 11.29 | 6.43 | 0.515 | 9.94 | 20.29 | 14.81 | 0.766 | 20.30 | 44.55 | 32.03 | 0.720 |
| Age30 | 4.75 | 10.03 | 5.67 | 0.505 | 11.14 | 22.41 | 16.47 | 0.781 | 22.31 | 47.93 | 35.29 | 0.755 |
| Age40 | 2.04 | 10.90 | 7.17 | 0.139 | 11.04 | 24.97 | 19.92 | 0.769 | 20.47 | 51.10 | 41.25 | 0.633 |

**Table S2.** Genetic and phenotypic coefficients of variation and heritability by age in Gunpo (GP)

|  | Height | | | | Diameter | | | | Volume | | | |  |
| --- | --- | --- | --- | --- | --- | --- | --- | --- | --- | --- | --- | --- | --- |
|  | *CV_G_* | *CV_P_* | *CV_E_* | *h^2^* | *CV_G_* | *CV_P_* | *CV_E_* | *h^2^* | *CV_G_* | *CV_P_* | *CV_E_* | *h^2^* |  |
| Age05 | 12.63 | 26.54 | 17.54 | 0.610 | 6.20 | 19.43 | 11.47 | 0.402 | 0.02 | 0.05 | 0.03 | 0.538 |  |
| Age08 | 10.87 | 31.60 | 21.78 | 0.469 | 6.96 | 25.83 | 17.43 | 0.288 | 0.29 | 1.05 | 0.71 | 0.299 |  |
| Age10 | 9.81 | 28.46 | 19.48 | 0.471 | 9.03 | 29.16 | 21.09 | 0.381 | 1.24 | 4.27 | 3.09 | 0.335 |  |
| Age15 | 7.64 | 21.20 | 12.54 | 0.475 | 11.16 | 31.05 | 20.76 | 0.511 | 7.87 | 23.54 | 15.52 | 0.443 |  |
| Age18 | 4.88 | 15.42 | 10.60 | 0.397 | 7.74 | 22.88 | 15.67 | 0.454 | 11.25 | 32.96 | 22.87 | 0.462 |  |
| Age20 | 3.81 | 11.92 | 8.30 | 0.406 | 6.90 | 21.11 | 15.25 | 0.424 | 11.45 | 33.65 | 24.24 | 0.459 |  |
| Age23 | 4.18 | 11.77 | 8.59 | 0.500 | 7.14 | 20.84 | 15.91 | 0.465 | 12.71 | 37.23 | 28.43 | 0.462 |  |
| Age26 | 3.37 | 10.29 | 6.94 | 0.426 | 7.70 | 19.67 | 14.63 | 0.606 | 14.56 | 38.98 | 29.11 | 0.552 |  |
| Age29 | 2.66 | 8.22 | 5.92 | 0.416 | 7.10 | 17.48 | 13.06 | 0.650 | 14.24 | 37.27 | 28.07 | 0.576 |  |
| Age30 | 2.63 | 8.34 | 6.24 | 0.395 | 7.29 | 17.76 | 13.18 | 0.664 | 14.78 | 37.69 | 28.11 | 0.607 |  |
| Age35 | 2.02 | 6.50 | 2.16 | 0.188 | 6.60 | 18.79 | 14.98 | 0.489 | 13.60 | 38.63 | 30.67 | 0.491 |  |
| Age40 | 1.39 | 9.67 | 5.55 | 0.083 | 6.90 | 19.97 | 16.22 | 0.472 | 13.96 | 42.94 | 35.10 | 0.419 |  |

**Table S3.** Phenotypic and genetic correlations of volume between Age 40 and other ages in the *Pinus koraiensis* progeny trial

|  | Phenotypic correlation | | Genetic correlation | | Spearman’s rank correlation | | |  |
| --- | --- | --- | --- | --- | --- | --- | --- | --- |
| Age | CJ | GP | CJ | GP | | CJ | GP | |
| 5 | - | 0.16 | - | 0.625 | | 0.412 | 0.443 | |
| 8 | 0.31 | 0.37 | 0.798 | 0.625 | | 0.695 | 0.552 | |
| 10 | 0.37 | 0.42 | 0.751 | 0.594 | | 0.614 | 0.482 | |
| 13 | 0.43 |  | 0.804 | - | | 0.759 | - | |
| 15 | 0.49 | 0.5 | 0.745 | 0.608 | | 0.716 | 0.563 | |
| 18 | 0.66 | 0.63 | 0.896 | 0.803 | | 0.799 | 0.703 | |
| 20 | 0.74 | 0.7 | 0.929 | 0.842 | | 0.826 | 0.775 | |
| 23 | 0.77 | 0.76 | 0.956 | 0.921 | | 0.900 | 0.848 | |
| 26 | 0.87 | 0.84 | 0.985 | 0.965 | | 0.944 | 0.877 | |
| 29 | - | 0.88 | - | 0.965 | | - | 0.890 | |
| 30 | 0.91 | 0.89 | 0.998 | 0.988 | | 0.954 | 0.945 | |
| 35 | - | 0.92 | - | 1.000 | | - | 0.965 | |
| 40 | 1 | 1 | 1 | 1.000 | | 1 | 1.000 | |
